# Identification of a weighted urinary microbial signature for bladder cancer discrimination

**DOI:** 10.3389/fonc.2026.1784501

**Published:** 2026-03-13

**Authors:** Filippo Russo, Lorella Tripodi, Filomena Caldora, Savio Domenico Pandolfo, Achille Aveta, Carmela Nardelli, Ciro Imbimbo, Sisto Perdonà, Lucio Pastore, Giuseppe Castaldo

**Affiliations:** 1 Dipartimento di Medicina Molecolare e Biotecnologie Mediche, Università degli Studi di Napoli Federico II, Naples, Italy; 2 CEINGE-Biotecnologie Avanzate Franco Salvatore, Naples, Italy; 3 Dipartimento di Medicina Clinica e Chirurgia, Università degli Studi di Napoli Federico II, Naples, Italy; 4 Dipartimento di Neuroscienze, Scienze Riproduttive e Odontostomatologiche, Università degli Studi di Napoli Federico II, Naples, Italy; 5 Dipartimento di Medicina Clinica, Sanità Pubblica, Scienze della Vita e dell’Ambiente, Università degli Studi dell’Aquila, L’Aquila, Italy; 6 Dipartimento di Urologia, Istituto Nazionale Tumori, IRCCS, “Fondazione G. Pascale”, Naples, Italy

**Keywords:** bacterial signature, bladder cancer, machine learning, urobiome, weighted composite index

## Abstract

**Introduction:**

Growing evidence from microbiome studies has demonstrated associations between dysbiosis and cancers, including bladder cancer (BCa). Our recent works on urobiome revealed a different microbial composition in BCa patients compared to controls. The aim of this work was to create a Weighted Composite Index (WCI) to distinguish BCa-affected patients (mBCa) from healthy controls (mHC) in a cohort of male aged over 50 years.

**Methods:**

Urobiome data from 51 subjects (27 mBCa and 24 mHC) were analyzed. Random Forest (RF) classifier was trained to identify genera and species which significantly contributed to discriminating between mBCa and mHC group. A weighted normalization approach was used to compute separate WCIs at genus and species levels and in-silico validation test were performed to assess the models’ robustness.

**Results:**

the WCI was calculated for each patient at both genera and species levels, showing a significant difference between the two groups (*p* < 0.0001) in both comparisons. WCIs showed superior discriminative performances compared to any individual taxon used for the model construction. Despite the need for validation in larger independent cohorts, the in-silico validation pipeline showed a stable high sensitivity of the models.

**Conclusions:**

Our findings identified a candidate urinary microbial signature in a biomarker discovery setting associated with bladder cancer. This hypothesis-generating approach may contribute to the identification of a non-invasive biomarker, which requires validation in larger, independent cohorts before clinical application.

## 1 Introduction

In recent years, the advent of next-generation sequencing (NGS) technologies had driven growing interest in the study of the urinary microbiome or urobiome, defined as the community of microorganisms that colonize the urinary tract. This interest arises from a revision of the long-standing paradigm that considered urine a sterile fluid (1). Despite challenges in studying the urobiome (low microbial biomass in urine, variability in sampling methods, and the influence of specific factors shaping the bladder microenvironment), several evidence have highlighted the correlation between urinary dysbiosis and several urinary tract disorders (2–4). Studies revealed an association between urinary dysbiosis and bladder cancer (BCa), demonstrating a decreased abundance of bacteria with proven protective effects (Lactobacillus) and the increase of bacteria already associated with other types of tumor (Fusobacterium, Streptococcus, and Pseudomonas) (5, 6). In this context, our previous studies demonstrated significant differences in the urinary bacterial profile of BCa patients compared to healthy subjects (7). Specifically, *Porphyromonas somerae* resulted significantly enriched in male BCa-affected patients aged over 50 years, compared to age- and sex-matched healthy controls. This increase was also further validated through digital droplet polymerase chain reaction (ddPCR) (8). Advances in urinary microbiome research have led to growing interest in developing predictive models that integrate urinary microbial composition data into interpretable diagnostic tools (9). Moreover, evidence on microbial urinary communities remain heterogeneous and frequently conflicting, especially due to methodological differences and the used bioinformatics pipelines (4). However, significant progress has been made in leveraging microbiome data to develop predictive tools that exploit microbial patterns to infer disease status (10). Gao et al. developed a predictive model for colorectal cancer based on shotgun metagenomic data of the gut microbiome, demonstrating how the integration of high-dimensional microbiome data can increase the predictive performance of statistical models, introducing the concept of a “microbiome signature” (11). Similarly, in a prospective study of women undergoing *in vitro* fertilization, Bar et al. developed a predictive model integrating vaginal microbiome data with inflammatory markers to estimate clinical pregnancy likelihood (12). These studies proved that the microbiome-based composite models have been successfully attempted in other body regions.

The present study was designed as a biomarker discovery analysis aimed at defining an interpretable urinary microbial signature associated with bladder cancer. To this end, we developed a quantitative composite index based on 16S rRNA sequencing data of the urobiome, constructed using a Random Forest–based feature selection and weighting strategy, and evaluated at both genus and species levels in men over 50 years of age.

## 2 Method

### 2.1 Study design

This study is a retrospective analysis of a subset of 51 male participants selected from the cohort enrolled between October 2021 and April 2023 at the Urology Clinic of the Federico II University Hospital in Naples for our previous study (7). Selection was restricted to men aged more than 50 years, given the higher incidence of bladder cancer in this demographic (13). Subjects were divided in two groups: 27 patients with a histological diagnosis of bladder urothelial carcinoma (mBCa group; mean age=73.1 ± 9.5) and 24 healthy volunteer male controls (mHC group; mean age=61.6 ± 7.6). Importantly, samples from the mBCa group were collected prior to any instrumentation or surgical procedure (e.g., TURBT procedure or insertion of an indwelling catheter). The mHC group consisted of healthy male volunteers enrolled during the same study period and from the same geographic area, and were not recruited among patients referred to the urology clinic for other complaints. To minimize technical variability, urine samples from both groups were collected using an identical protocol: participants provided a first-morning urine sample in a sterile container, including the first stream to increase biomass recovery. Inclusion criteria for both mBCa and mHC groups included absence of urinary tract infections at the time of urine collection, no history of decompensated diabetes, and no antibiotic treatment in the previous month. Exclusion criteria applied to reduce microbiome alterations included the presence of indwelling catheters, nephrostomy tubes, or urinary diversions. Additionally, patients who had received intravesical therapy (e.g., BCG or chemotherapy) were not included in this cohort. Clinical characteristics of the subjects included in BCa group are reported in **Supplementary Materials** (**Supplementary Table S1**). Urinary microbiome analysis was conducted, as indicated in the previous study, using 16S rRNA gene sequencing (V3–V6 regions), with the Microbiota Solution B kit (Arrow Diagnostics, Genoa, Italy) (7). Sequencing libraries were prepared following standard protocols, including quality control and contamination assessment with negative controls.

### 2.2 Data processing

The raw sequencing data obtained were exported as Fastq files and aligned using MicrobAT bioinformatics software (Suite-SmartSeq, Novara, Italy). Operational Taxonomic Units (OTUs) were assigned to the sequences of each sample by comparison with the Ribosomal Database Project (RDP) reference database. All sequences identified as “unclassified Archea” were removed from the OTUs. *Propionibacterium acnes*, *Sphingomonas* sp. *Oral clone AV069* and Ralstonia taxa, corresponding to known laboratory contaminants, were removed based on their presence in negative control samples (14, 15). Following this initial filtering, the resulting OTU table was uploaded into the MicrobiomeAnalyst platform (https://www.microbiomeanalyst.ca) for further processing (16). Specifically, we used the Marker Data Profiling module to process and analyze the taxonomic count data derived from the OTU table. In this module, additional filtering steps were included: low count filter (taxa that did not reach a minimum of 20 counts in at least 10% of the samples were excluded) and low variance filter (taxa with a variance below the 10th percentile based on the interquartile range were excluded). Sequencing depth distribution was evaluated to assess potential bias due to uneven sampling effort (read counts per group were reported in **Supplementary Figure S1**). No rarefaction was applied to avoid the loss of valid sequencing information, particularly for low-abundance taxa potentially relevant for biomarker discovery. Filtered OTU data were exported as absolute abundances (AA; counts) and subsequently converted into relative abundances (RA, %) using total sum scaling (TSS) normalization for downstream analyses (**Supplementary Figure S2**).

### 2.3 Weighted composite index development

A Weighted Composite Index (WCI) was developed to quantify the differential distribution of taxa’s RAs associated with mBCa and mHC. This approach aimed to identify discriminating bacterial taxon by using supervised machine learning and integrate them into a continuous composite score. To this end, RAs were analyzed separately at the genus and species taxonomic levels. Given the biomarker discovery aim of this study and the absence of an independent validation cohort, feature selection and weight derivation were performed once on the full dataset to define an interpretable microbial signature. The Random Forest (RF) algorithm was applied on the entire dataset to classify the variables entered into the model (17). The algorithm parameters were set to 1500 trees (*ntree* = 1500), and the number of trees was increased until out-of-bag (OOB) error and variable importance estimates stabilized. The number of variables tested at each split (*mtry*) was set to the square root of the total number of features (≈ $\sqrt{f}$), resulting in *mtry* = 5 for genera ( f=33 variables) and *mtry* = 9 for species ( f=85 variables). For each taxonomic level, the OOB error was measured as the prediction error and the Mean Decrease Accuracy (MDA) of each taxon was calculated. Subsequently, only taxa with MDA>5 were selected. To standardize taxa’s contribution to our final model, the MDAs associated with each variable were normalized and weighted. Normalization was applied by calculating the ratio between each MDA value and the total sum of MDAs in the corresponding group, resulting in normalized MDA (nMDA) value. Two sub-scores were then defined: the BCa-related sub-score, derived from the sum of the relative abundances of the discriminating taxa for the mBCa group; and the HC-related sub-score, calculated similarly but for the taxa discriminating for the mHC>50y group. The final WCI index was obtained by subtracting the two partial scores, according to the following formula.


$$WCI=\left[\sum_{\text{i}\in T_{mBCa}}\left(RA_{i}\cdot nMDA_{i}\right)\right]-\left[\sum_{\text{j}\in T_{mHC}}\left(RA_{j}\cdot nMDA_{j}\right)\right]$$

where:

- $T_{mBCa}$= BCa-associated Taxa, discriminant taxa for the BCa group;
- $T_{mHC}$= HC associated Taxa, discriminant taxa for the HC group;
- i = any variable belonging to the T_mBCa_;
- j = any variable belonging to the T_mHC_;
- RA = relative abundance of a specific taxon;
- nMDA = normalized Mean Decrease Accuracy, representing the weight assigned to the variable, based on its normalized Mean Decrease Accuracy.

This method allowed us to create a continuous index that reflects an increase in BCa-associated bacteria (when the value shown is positive) or an increase in HC-associated bacteria (when the value shown is negative).

### 2.4 Statistical analysis

Mann-Whitney test was performed to assess significant differences between the two analyzed groups. To evaluate the model performance, Receiver Operating Characteristic (ROC) analyses were performed on WCI results, reporting the Area Under the Curve (AUC), sensitivity, specificity, and 95% confidence intervals (95% CI, Wilson/Brown method). For ROC analyses optimal cut-off values were determined using the Youden index. To assess the potential confounding effect of age, a multivariate logistic regression analysis was performed, including age and WCI as covariates. In addition, ROC analyses were also conducted for each individual discriminant taxon, allowing comparison of WCI against each single bacterial feature. Moreover, a repeated 5-fold cross-validation (CV) strategy was conducted to evaluate the discriminatory behavior and internal consistency of the previously calculated WCIs. Results of CVs were reported as mean AUC, mean sensitivity, mean specificity, and 95% CI (calculated based on the t-distribution of the 10 repetitions). Furthermore, a permutation test with 1000 iterations was performed. The *p-value* was calculated as the proportion of permutations with a random AUC greater than or equal to the AUC observed in the repeated 5-fold cross-validation. All computational analyses for the construction and validation of the WCI were conducted with RStudio Version 2024.12.0 + 467 software (Posit PBC, Boston, MA), using the randomForest, Hmisc, corrplot, caret, and ggplot2 libraries. All statistical analyses and graphs were processed with GraphPad PRISM 10, version 10.1.1 (GraphPad Software Inc., Boston, MA).

## 3 Results

### 3.1 Identification of discriminant taxa using random forest algorithm

The RA data of mBCa and mHC groups were examined using the RF algorithm, in order to classify the variables at the genus (**Supplementary Table S2**) and species (**Supplementary Table S3**) levels. At the genus level, the number of variables tested with the algorithm was 33, with mtry=5 and ntree=1500. At this taxonomic level, OOB error was 31.37%. At the species level, 85 variables were examined, with mtry=9 and ntree=1500, with an OOB of 21.57%. Taxa with MDA>5 were listed in decreasing order in **Table 1**: they represented the variables with the greatest contribution to the predictive ability between the mBCa and mHC groups at both genus and species levels.

**Table 1:** Top discriminatory taxa ranked by contribution to prediction (mean decrease accuracy).

Taxon	MDA	nMDA	mBCa (RA %)	mHC (RA %)	Mann-Whitney (*p-value*)
Genus					
Enhydrobacter	13.68	0.20	0.05	1.77	*0.005*
Porphyromonas	12.65	0.18	6.05	0.28	*0.001*
Arthrobacter	9.77	0.14	0.01	2.78	*0.001*
Aerococcus	9.32	0.13	3.00	0.26	*0.003*
Enterobacter	8.84	0.13	0.02	0.10	*0.029*
Sphingomonas	8.55	0.12	0.00	0.22	*0.009*
Anaerococcus	7.22	0.10	3.97	0.87	*0.005*
Species					
*Porphyromonas somerae*	11.45	0.14	2.34	0.04	*<0.001*
*Moraxella osloensis*	9.85	0.12	0.02	0.78	*0.004*
*Anaerococcus* sp *gpac028*	9.75	0.12	0.20	0.02	*<0.001*
*Aerococcus urinae*	9.48	0.12	3.00	0.26	*0.003*
*Porphyromonas* sp *2007b*	8.08	0.10	0.95	0.01	*<0.001*
*Arthrobacter* sp. *mb182*	7.39	0.09	0.01	2.78	*0.001*
*Endosymbiont of Sphenophorus levis*	6.68	0.08	0.02	0.10	*0.029*
*Gamma-proteobacterium St.07B*	6.53	0.08	0.02	0.70	*0.023*
*Actinobaculum massiliense*	6.29	0.08	0.03	1.88	*0.037*
*Porphyromonas asaccharolytica*	5.52	0.07	1.26	0.09	*0.003*

MDA, Mean Decrease Accuracy; nMDA, normalized Mean Decrease Accuracy; RA, Relative Abundance.

### 3.2 Weighted composite index in mBCa and mHC groups

**Figure 1:**
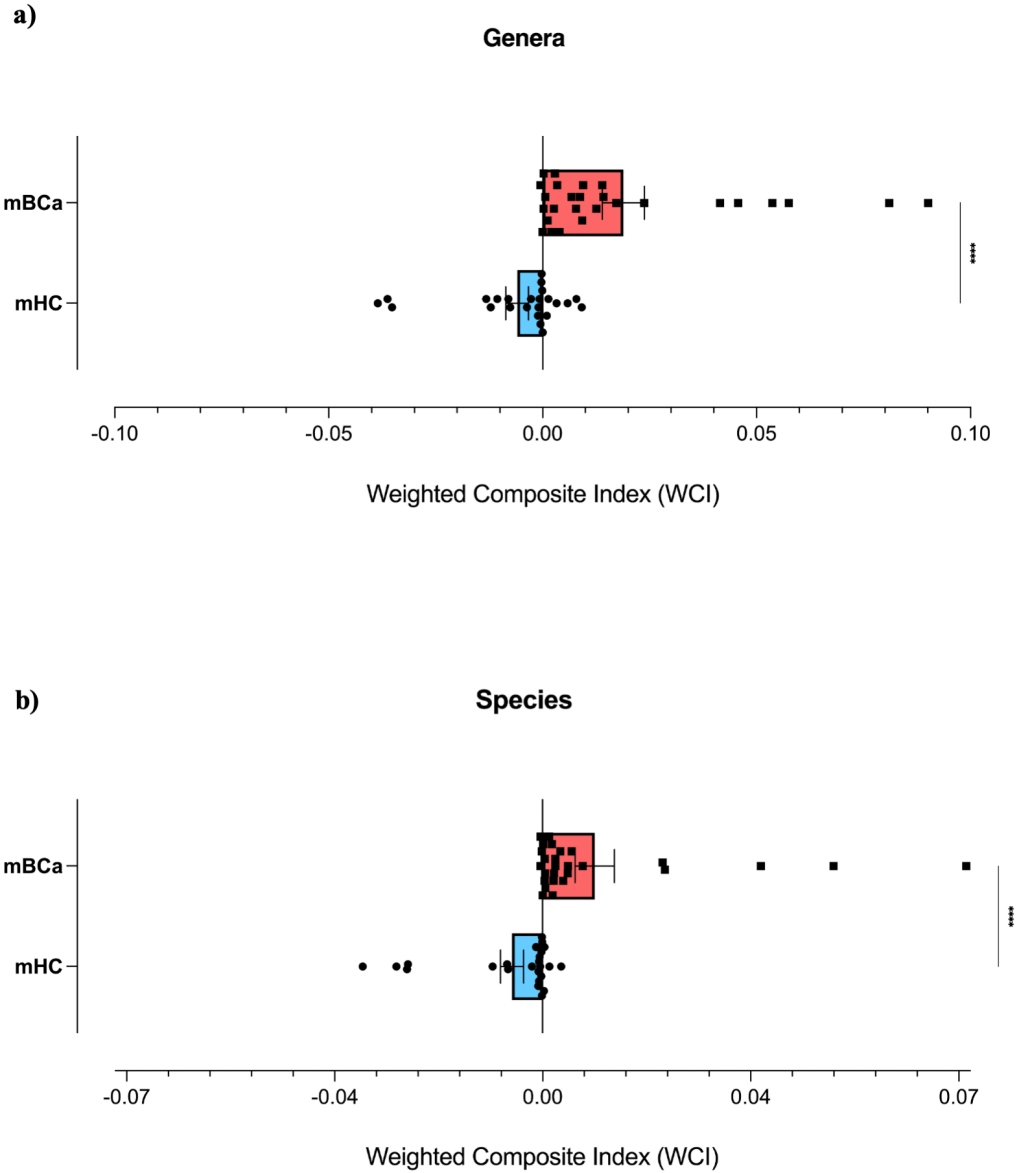
Distribution of the weighted composite index (WCI) in the mBCa and mHC groups. The scatter plot with bars shows the distribution (mean with SEM) of WCIs calculated at the genus **(a)** and species **(b)** levels in the comparison between the mBCa (black squares and red bars) and mHC (black circles and blue bars) groups. On the right, the statistically significant difference is represented (Mann-Whitney test, *p* < 0.0001 in both comparisons). The symbol “****” indicates p < 0.0001 (Mann–Whitney test).

According to the formula described in paragraph 2.3, we attributed the selected taxa to the mBCa or mHC group, basing on the highest mean RA value between the two groups. Then, the WCI was calculated for each patient at both the genus and species levels. For the genus taxonomic level, the mean WCI calculated in the mBCa group gave a value of 0.019 ± 0.025, while in the mHC it gave a value of -0.006 ± 0.013 (**Figure 1a**). At the species level, the mean WCI calculated in the mBCa group was 0.009 ± 0.017, while in the mHC group it showed a value of -0.005 ± 0.0095 (**Figure 1b**). At both taxonomic levels, the comparison between the two groups showed a statistically significant difference in the calculated WCI (Mann-Whitney test, *p* < 0.0001 in both comparisons). These results indicate that positive WCI values reflect a higher abundance of BCa-associated taxa, whereas negative values reflect a higher abundance of HC-associated taxa.

### 3.3 Performance evaluation of weighted composite index

**Figure 2:**
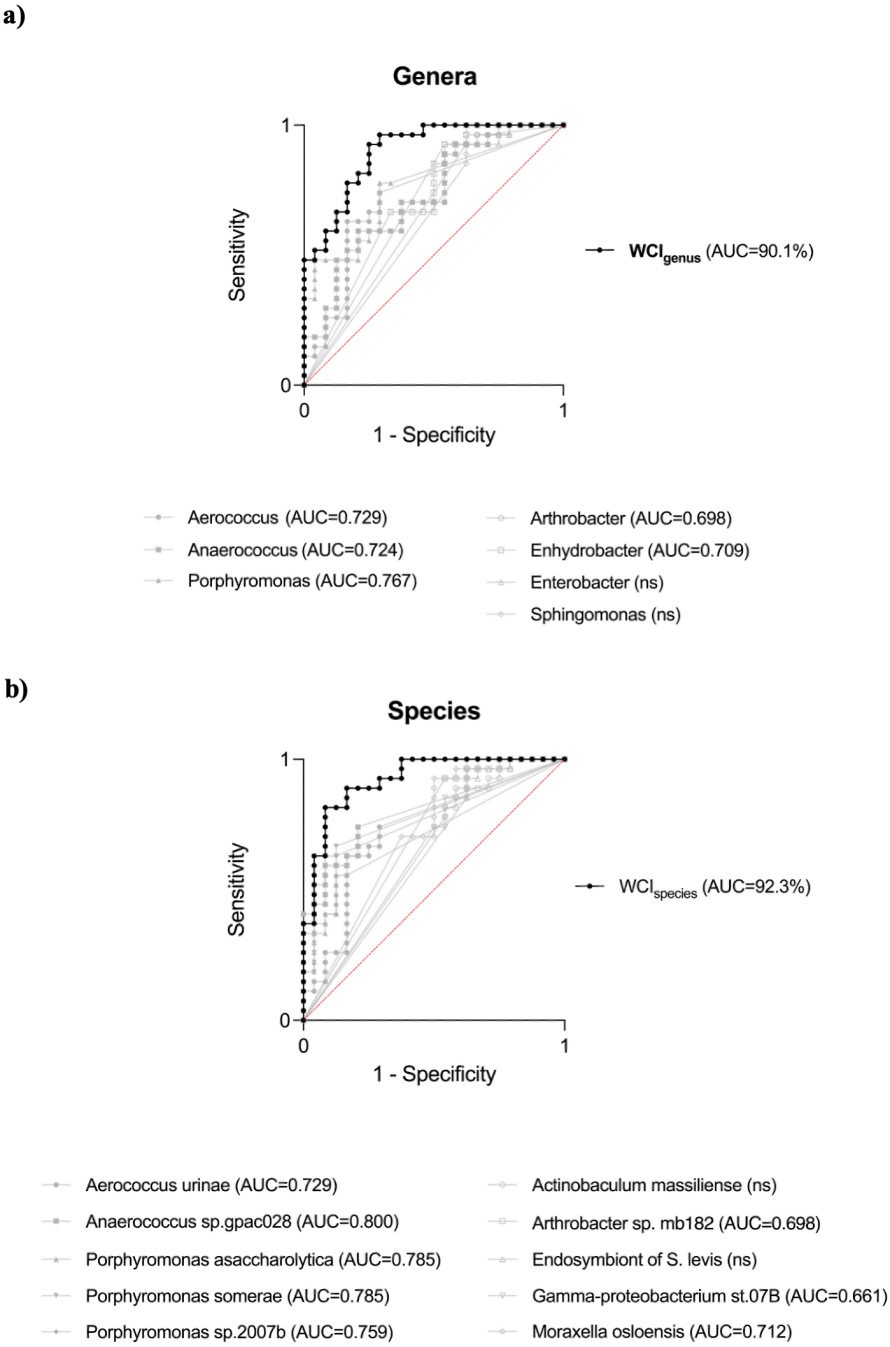
ROC curve analysis of the weighted composite index (WCI) and individual taxa. ROC curves generated for the genus-level and species-level WCIs and single taxon. **(a)** WCI_genus_ (black curves) showed an AUC of 90.1% (95% CI = 81.9–98.2%; *p* < 0.0001), outperforming all single genera (grey curves): Porphyromonas, AUC = 76.7%; Aerococcus, AUC = 72.9%; Anaerococcus, AUC = 72.4%, Arthrobacter, AUC = 69.8%; Enhydrobacter, AUC = 70.9%; the genera Enterobacter and Sphingomonas did not reach statistical significance (ns). **(b)** WCI_species_ achieved an AUC of 92.3% (95% CI = 85.1–99.5%; *p* < 0.0001). AUC of all single species resulted lower: *Porphyromonas asaccharolytica*, AUC = 78.5%; *Aerococcus urinae*, AUC = 72.9%; *Porphyromonas* sp. *2007b*, AUC = 75.9%; *Porphyromonas somerae*, AUC = 78.5%; *Anaerococcus* sp. *gpac028*, AUC = 80.0%; *Gamma-proteobacterium st.07B*, AUC = 66.1%; *Arthrobacter* sp. *mb182*, AUC = 69.8%; *Moraxella osloensis*, AUC = 71.2%; the *Endosymbiont of Sphenophorus levis* and *Actinobaculum massiliense* species did not reach statistical significance (ns). Filled symbols indicate taxa with higher relative abundance in the mBCa group, while open symbols indicate taxa more abundant in the mHC group.

To assess the WCI’s ability to discriminate between mBCa and mHC, ROC curves analyses were performed. Separate ROC curves were created for the genus-level WCI (WCI_genus_) and species-level WCI (WCI_species_). Furthermore, the AUCs of the ROC curves of the individual variables used to create the two indices were calculated to compare the performance of the index with that of individual genera or species. At the genus level, using WCI_genus_, the ROC curve showed an AUC of 90.1% (95% CI = 81.9–98.2%; *p* < 0.0001), higher than the ones obtained for individual bacterial genera (**Figure 2a**). Furthermore, the WCI_genus_ performance metrics showed a sensitivity of 92.6% (95% CI = 76.6–98.7%) with a specificity of 75.0% (95% CI = 55.1–88.0%). The ROC curve of WCI_species_ showed an AUC of 92.3% (95% CI = 85.1–99.5%; *p* < 0.0001), with a sensitivity of 81.5% (95% CI = 63.3–91.8%) and a specificity of 91.7% (95% CI = 74.2–98.5%). The AUC of the WCI_species_ was higher than the AUCs of all individual species included in the WCI_species_ model (**Figure 2b**). Given the age difference between the mBCa and mHC groups (as reported in section 2.1), we performed two multivariate logistic regressions to evaluate whether the WCI_genus_ and WCI_species_ predictive values were driven by age. The age-adjusted analyses confirmed that both WCIs remained significant independent predictors at both genus (*p* = 0.012) and species (*p* = 0.018) levels.

### 3.4 *In-silico* validation of weighted composite index

A two-step in-silico pipeline was performed to validate the indices: the 10-times repeated 5-fold Cross-Validation, to estimate the reliability of the WCI, and the Permutation Test with 1000 iterations, to compare the validation performance with that expected from possible random models. This pipeline was designed to mitigate the lack of an independent validation cohort, the main limitation of this study.

**Figure 3:**
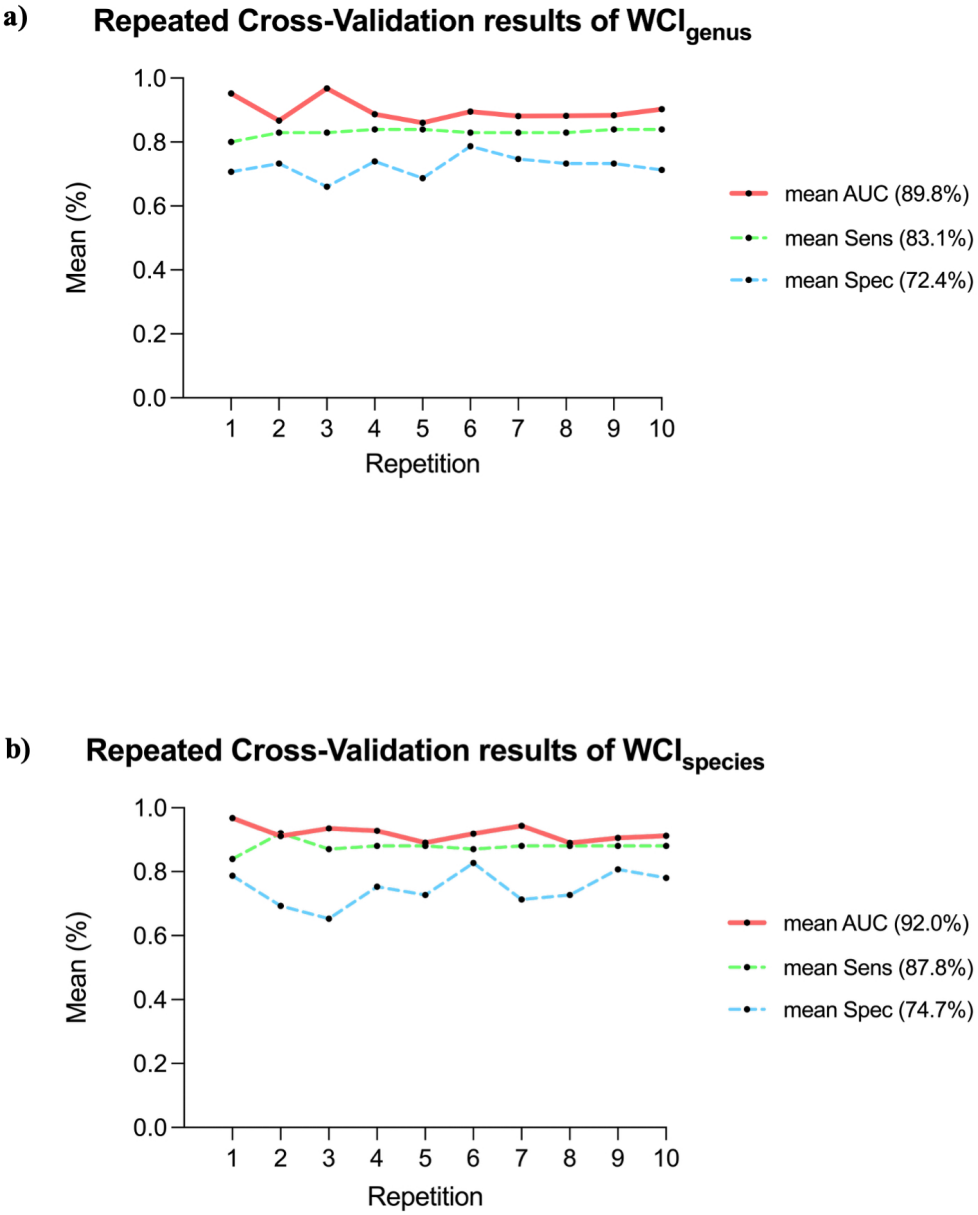
Repeated cross-validation results of the weighted composite index (WCI). **(a)** Performance metrics of WCI_genus_ across a 10-times repeated 5-fold cross-validation. The average AUC was 89.8% (95% CI = 87.3–92.3%; min-max range: 86.0-96.8%, red solid line), with average sensitivity 83.1% (95% CI = 82.2–84.0%; min-max range: 80.0–84.0%, green dashed line) and average specificity 72.4% (95% CI = 69.9–74.9%; min-max range: 66.0–78.7%, blue dashed line), respectively. **(b)** Performance metrics of WCI_species_ across the same cross-validation scheme. The average AUC was 92.0% (95% CI = 90.3–93.8%; min-max range: 88.9–96.8%, red solid line), with a mean sensitivity of 87.8% (95% CI = 86.4–89.2%; min-max range: 84.0–92.0%, green dashed line) and a mean specificity of 74.7% (95% CI = 70.8–78.5%; min -max range: 65.3–82.7%, blue dashed line).

In the WCI_genus_, the cross-validation resulted in an average AUC of 89.8% (95% CI = 87.3–92.3%), with average sensitivity and specificity of 83.1% (95% CI = 82.2–84.0%) and 72.4% (95% CI = 69.9–74.9%), respectively (**Figure 3a**). Across the 10 repetitions, the WCI_genus_ consistently achieved an AUC above 86%, with stable sensitivity values and slightly more variable specificity (ranging from 66% to 78.7%). The permutation test showed that this validation significantly outperformed the random models (*p* = 0.001). Similarly, WCI_species_ showed high stability, with an average AUC of 92.0% (95% CI = 90.3–93.8%) and all AUC values above 88.9%. The sensitivity remained stable, with an average of 87.8% (95% CI = 86.4–89.2%), while specificity was slightly more variable across repetitions (min-max range: 65.3–82.7%; average 74.7%; 95% CI = 70.8–78.5%) (**Figure 3b**). The permutation test showed a statistically significant difference from the average of the 1000 random iterations (*p* = 0.001). These trends confirmed the robustness of both indices, particularly WCI_species_, which displayed superior average performance and lower fluctuation in cross-validation metrics.

## 4 Discussion

In recent years, the study of the urinary microbiome has generated enormous interest, demonstrating the disease-associated shifts in urinary microbial composition (4). In our previous studies, we characterized the urobiome of BCa patients, identifying *P. somerae* as putative biomarker of bladder cancer (7, 8, 18). The present study aimed to develop a microbiome-based composite index, trained through RF algorithm, to capture the microbial imbalance between BCa and HC group. By reprocessing 16S-rRNA gene sequencing data, discriminating taxa were subsequently integrated into a combined index, capable of synthesizing in a single continuous parameter the urinary microbiome imbalance towards a BCa- or HC-associated microbial profiles. The analysis started with the selection of taxa at the genus and species levels. Then, the taxa selected through classification, weighted based on their discriminatory ability, were integrated into the respective WCIs at the specific taxonomic level. Both WCI_genus_ and WCI_species_ effectively distinguished BCa from HC subjects, with a high predictive performance superior to that obtained from the individual biomarkers.

At the genus level, the most represented bacteria in the mBCa group have already been described in the literature in association with pathological conditions (6, 19). Anaerococcus has been detected in greater abundance in patients with bladder cancer and is also implicated in other urinary tract pathologies. Aerococcus is known to be present in chronic urinary tract infections, while Porphyromonas is often associated with persistent inflammatory states. These observations suggest a possible role for these taxa in modulating the tumor microenvironment. Conversely, the most abundant genera in the mHC group had also been reported in the literature in different pathological contexts (6): Enterobacter has been associated with stress urinary incontinence; Arthrobacter has been found in subjects with urge incontinence; Sphingomonas has also been detected in the tumor tissue of patients with BCa. These findings highlighted how the presence or absence of specific bacterium is not sufficient to define its pathogenic or protective role. The relative variation of the overall imbalance in the bacterial composition more likely provides a reliable indication of a pathological or physiological state, supporting the use of composite-score approach to identify a microbial signature related to the disease. Remarkably, WCI showed a higher predictive capacity than the one of the single biomarkers alone, as illustrated. To support the robustness of the model, the performance of WCI was further *in-silico* validated through a 10-time repeated 5-fold cross-validation procedure and a permutation test with 1,000 iterations. In particular, cross-validation was used to estimate the stability of the index by simulating a wide variety of samplings. In particular, the analysis showed that the WCI_species_ had an average sensitivity higher than its specificity (87.8% *vs*. 74.7%), reversing the trend observed in the ROC curve analysis (81.5% *vs*. 91.7%). This observation suggests that, in a hypothetical realistic scenario, the WCI_species_ may be particularly suited to identify true positive cases, a feature that could be relevant for future screening-oriented applications. Beyond diagnostic discrimination, a recent study in non-muscle invasive bladder cancer patients reported an association between higher urinary microbiome diversity after TURBT and increased recurrence risk (20), suggesting that urinary microbiome-derived metrics could potentially contribute to future risk stratification, pending dedicated validation studies.

This study has some limitations. First, the small sample size could introduce selection bias. In addition, our study focused on men aged >50 years, and consequently, the performance of the WCI in women or younger men remains unknown. Given the reported sex-specific differences in urinary microbiome composition between female and male BCa patients (21), future validation studies should incorporate sex-stratified analyses to more comprehensively capture the complexity of microbiome–cancer associations. Moreover, the mBCa group was older than the control group (73.1 *vs* 61.6 years), which represents a potential confounder (22). In fact, aging is frequently associated with conditions such as benign prostatic hyperplasia (BPH) and lower urinary tract symptoms (LUTS), which have been shown to influence urinary microbial composition (23, 24). To address this concern, we performed an age-adjusted multivariable analysis, which suggested that the identified signatures captured disease-specific signals beyond aging-associated shifts. Moreover, other host-related covariates known to influence the urobiome (e.g., BMI and smoking status) were not consistently available for the entire retrospective cohort, particularly among controls. Therefore, residual confounding cannot be fully excluded, and future prospective validation studies should systematically collect relevant urological and non-urological comorbidities and lifestyle factors. Recent evidence has also highlighted that urinary and tumor-associated microbiome profiles can be influenced by disease activity and treatment status, including intravesical therapies, as demonstrated by differences reported between responders and non-responders to Bacillus Calmette-Guérin (BCG) treatment (25). In this context, we deliberately restricted our analysis to treatment-naïve patients at the time of urine collection to minimize therapy-related confounding, while acknowledging that future prospective studies should further stratify patients according to disease status and treatment exposure. Furthermore, although a repeated cross-validation strategy and permutation testing were used to assess internal robustness, internal validation does not replace validation in independent external cohorts. Accordingly, the reported performance metrics should be interpreted as reflecting within-cohort discrimination and internal consistency of a pre-defined microbial signature, rather than unbiased estimates of generalizable predictive accuracy, which will require independent external validation. In this regard, a change in positive and negative predictive values ​​derived from this case-control cohort might be expected in real-world clinical settings (e.g., hematuria clinics, population-based screening scenarios, etc.).

In conclusion, in this study we developed a composite index capable of representing the urobiome imbalance of BCa-affected patients, through a data-driven analysis focused on the entire microbial community. The WCI was calculated at both the genus and species levels, demonstrating high discriminatory power between the two groups analyzed. Results obtained from cross-validation procedures and permutation tests support the model’s robustness, despite the lack of an independent validation cohort, which remains necessary to confirm the diagnostic utility of the identified WCIs. Overall, we propose this composite index as a candidate, non-invasive urinary microbial signature associated with bladder cancer. This approach should be considered as hypothesis-generating and requires validation in larger and independent cohorts before any potential application in screening or risk stratification settings.

## Data Availability

Publicly available datasets were analyzed in this study. This data can be found here: https://www.ncbi.nlm.nih.gov/bioproject/?term=prjna981420 (Accession: PRJNA981420; ID: 981420).
