# Supplementary material for: Identification of a weighted urinary microbial signature for bladder cancer discrimination
Source: Front Oncol. 2026 Mar 13;16:1784501. doi: 10.3389/fonc.2026.1784501 (PMC13021399; doi:10.3389/fonc.2026.1784501)
Supplement: Supplementary file 1 [file DataSheet1.docx]

Supplementary Material


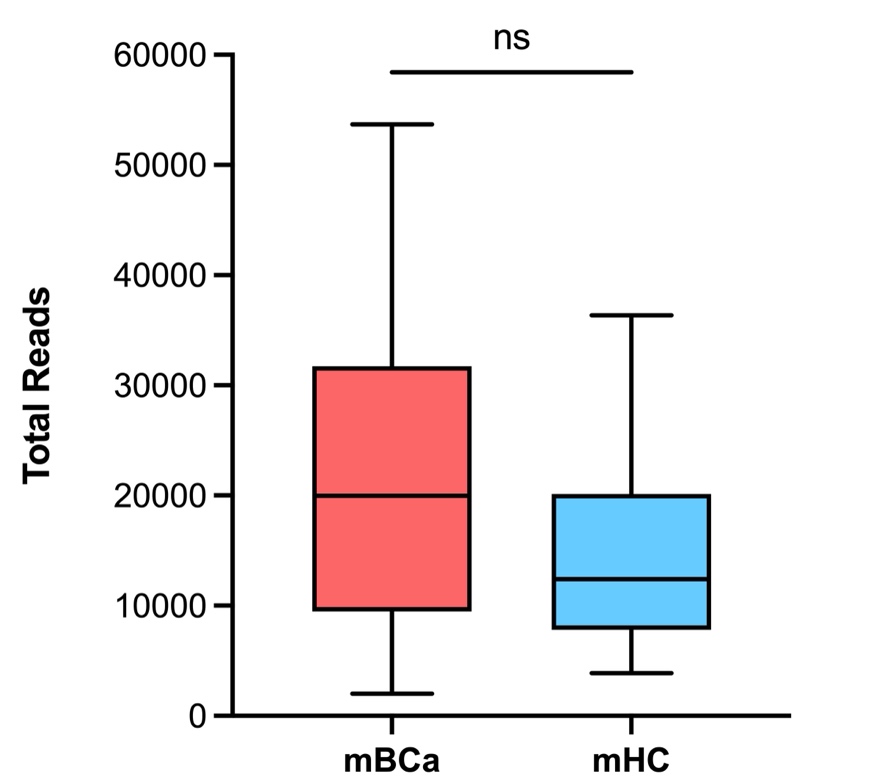


***Figure S1.*** **Distribution of sequencing depth across study groups.** The box plots illustrated the distribution of total sequencing reads (library size) in the male Bladder Cancer (mBCa, red box; n=27) and male Healthy Control (mHC, blue box; n=24) groups. The central line within each box represented the median value (mBCa median = 19,968 reads; mHC median = 12,395 reads), while the box edges defined the interquartile range (IQR, 25th to 75th percentiles). Whiskers represented the minimum and maximum values. No statistically significant difference in sequencing depth was observed between the two groups (Mann-Whitney U test, *p*=0.161), indicating a comparable sampling. Given the comparable sequencing depth observed between study groups, the use of relative abundance–based analyses without rarefaction were considered appropriate for the aims of the study.


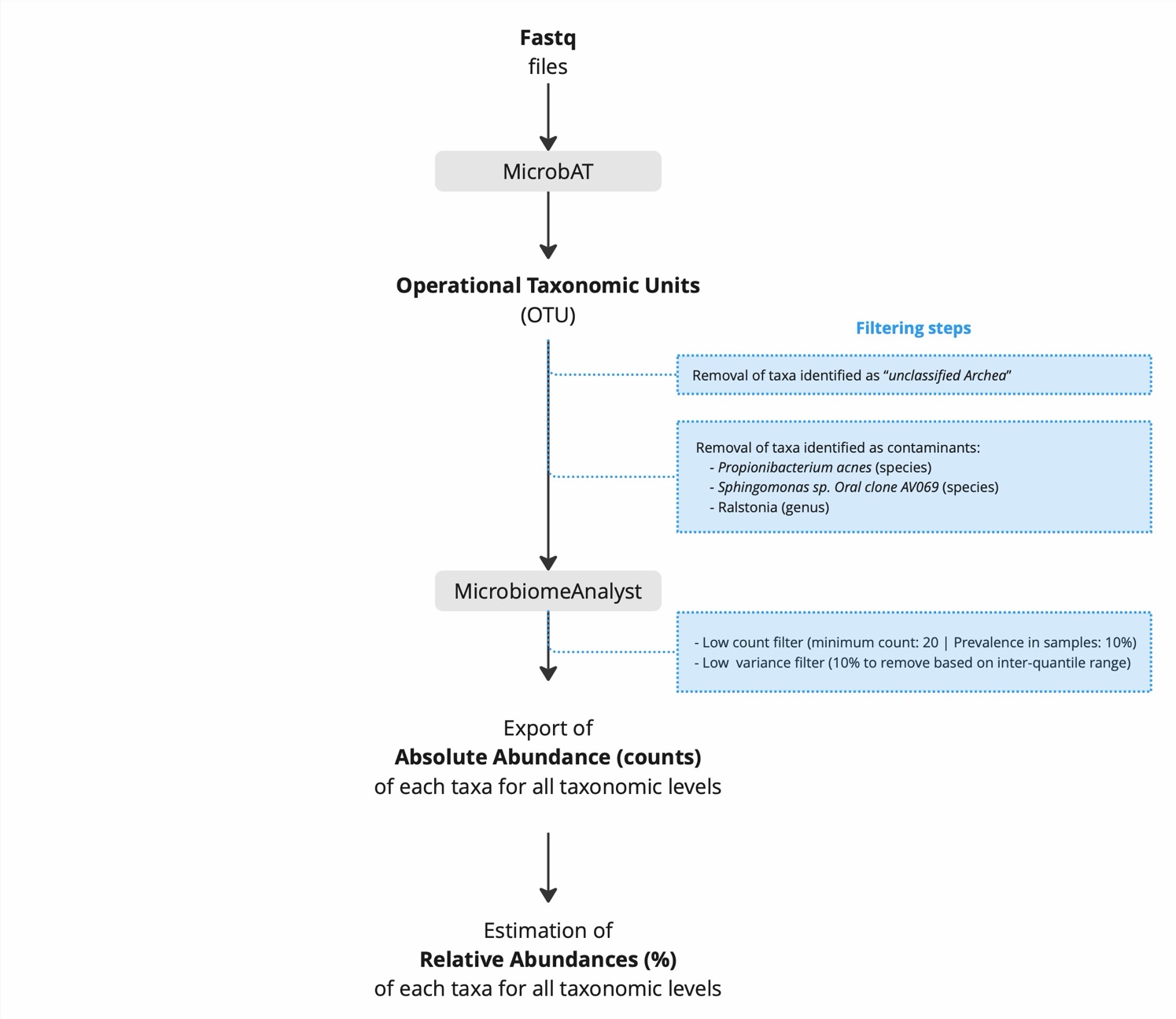


***Figure S2.*** **Workflow for processing microbiome sequencing data.** The figure illustrated the workflow used to process urinary 16S rRNA sequencing data, starting from raw Fastq files and ending with the calculation of relative taxonomic abundances (expressed as percentages). Negative controls consisted of sterile water blanks samples. They were included in each sequencing run (n=13 in total) and processed alongside biological samples. Analyses of these controls revealed the presence of known reagent- and laboratory-associated contaminants (14,15), including *Propionibacterium acnes*, *Sphingomonas* *sp. Oral clone AV069*, and Ralstonia spp., which were therefore removed from downstream analyses. All filtering steps are highlighted in light blue. Figure created using miro.com.

***Table S1.* Clinical characteristics of BCa-affected subjects included in the study**

| **Group** | **n** | **Age** | **BMI** | **Tumor characteristics** | **Tobacco** | **Alcohol** | **DM** | **HBP** |
| --- | --- | --- | --- | --- | --- | --- | --- | --- |
|  |  | (years ± SD) | (Kg ± SD) |  | (consumers) | (consumers) | (affected) | (affected) |
| **mBCa** | 27 | 73.1±9.5 | 27.3±3.3 | ***Bladder Cancer***  ***TNM***  Ta 9  Tis -  T1 11  T2 2  T2a 2  T2b 1  n.a. 2  ***Grading***  Gx -  G1 6  G3 19  n.a. 2 | 7 | 4 | 8 | 13 |

Abbreviations: mBCa=male bladder cancer-affected patients; n=numbers of subjects; SD=standard deviation; BMI=body mass index; DM=diabetes mellitus; HBP= High Blood Pressure.

***Table S2.* Relative abundance of genera in mBCa and mHC groups**

| **Genus** | **mBCa** | **mHC** |
| --- | --- | --- |
|  | *mean RA (%)* | *mean RA (%)* |
| Escherichia Shigella | 3.58 | 15.11 |
| Prevotella | 9.10 | 8.77 |
| Peptoniphilus | 8.00 | 3.47 |
| Streptococcus | 2.57 | 6.29 |
| Actinobaculum | 3.04 | 3.37 |
| Porphyromonas | 6.05 | 0.28 |
| Anaerococcus | 3.97 | 0.87 |
| Enterococcus | 0.86 | 3.78 |
| Finegoldia | 3.59 | 1.04 |
| Propionimicrobium | 3.20 | 1.28 |
| Aerococcus | 3.00 | 0.26 |
| Corynebacterium | 2.12 | 1.09 |
| Dialister | 1.22 | 1.64 |
| Arthrobacter | 0.01 | 2.78 |
| Campylobacter | 1.78 | 0.92 |
| Mobiluncus | 1.68 | 0.71 |
| Anaerosphaera | 1.12 | 1.12 |
| Veillonella | 0.81 | 1.03 |
| Enhydrobacter | 0.05 | 1.77 |
| Hallella | 0.60 | 1.00 |
| Negativicoccus | 1.05 | 0.42 |
| Fusobacterium | 0.47 | 0.98 |
| Propionibacterium | 0.60 | 0.74 |
| Staphylococcus | 0.45 | 0.43 |
| Actinomyces | 0.34 | 0.44 |
| Arcanobacterium | 0.17 | 0.06 |
| Sphingomonas | 0.00 | 0.22 |
| Howardella | 0.06 | 0.15 |
| Facklamia | 0.19 | 0.02 |
| Fastidiosipila | 0.12 | 0.06 |
| Lactovum | 0.04 | 0.13 |
| Enterobacter | 0.02 | 0.10 |
| Unclassified genus | 32.85 | 39.63 |

Abbreviations: **RA**=Relative Abundance

***Table S3.* Relative abundance of species in mBCa and mHC groups**

| **Species** | **mBCa** | **mHC** |
| --- | --- | --- |
|  | *mean RA (%)* | *mean RA (%)* |
| Escherichia coli | 1.44 | 6.68 |
| Prevotella timonensis | 4.21 | 1.58 |
| Peptoniphilus sp 2002 2300004 | 3.73 | 1.85 |
| Prevotella bivia | 0.79 | 4.37 |
| Enterococcus faecalis | 0.86 | 3.78 |
| Finegoldia magna | 3.09 | 0.90 |
| Propionimicrobium lymphophilum | 2.62 | 0.88 |
| Streptococcus anginosus subsp null | 1.24 | 2.08 |
| Streptococcus mitis | 0.13 | 3.17 |
| Aerococcus urinae | 3.00 | 0.26 |
| Arthrobacter sp mb182 | 0.01 | 2.78 |
| Swine fecal bacterium RF2B Pec14 | 0.19 | 2.59 |
| Campylobacter ureolyticus | 1.72 | 0.88 |
| Anaerococcus lactolyticus | 2.02 | 0.48 |
| Porphyromonas somerae | 2.34 | 0.04 |
| Shigella flexneri 2002017 | 0.83 | 1.47 |
| Actinobaculum schaalii | 1.52 | 0.73 |
| Prevotella disiens | 1.67 | 0.43 |
| Actinobaculum massiliense | 0.03 | 1.88 |
| Veillonella sp oral clone VeillE4 | 0.81 | 1.03 |
| Dialister propionicifaciens | 0.85 | 0.99 |
| Actinobaculum urinale | 1.07 | 0.52 |
| Mobiluncus curtisii | 1.08 | 0.39 |
| Fusobacterium nucleatum subsp null | 0.47 | 0.98 |
| Prevotella corporis | 0.25 | 1.15 |
| Porphyromonas asaccharolytica | 1.26 | 0.09 |
| Streptococcus pneumoniae | 1.13 | 0.20 |
| Corynebacterium sp 31595 | 1.03 | 0.29 |
| Peptoniphilus sp S9 AA1 4 | 0.87 | 0.36 |
| Actinobacterium PI GH2 1 C2 | 0.53 | 0.67 |
| Negativicoccus succinicivorans | 0.88 | 0.30 |
| Peptoniphilus lacrimalis | 0.92 | 0.23 |
| Bacterium CSR 63 | 0.08 | 0.93 |
| Porphyromonas sp 2007b | 0.95 | 0.01 |
| Prevotella sp S4 10 | 0.58 | 0.38 |
| Peptoniphilus sp BV3AC2 | 0.67 | 0.26 |
| Staphylococcus epidermidis | 0.45 | 0.43 |
| Moraxella osloensis | 0.02 | 0.78 |
| Corynebacterium pseudogenitalium | 0.44 | 0.36 |
| Prevotella buccalis | 0.58 | 0.15 |
| Gamma proteobacterium S St (0) 7B | 0.02 | 0.70 |
| Escherichia fergusonii | 0.05 | 0.63 |
| Anaerococcus prevotii DSM 20548 | 0.62 | 0.06 |
| Actinomyces turicensis | 0.23 | 0.42 |
| Dialister micraerophilus | 0.17 | 0.48 |
| Corynebacterium tuberculostearicum | 0.40 | 0.16 |
| Peptoniphilus duerdenii | 0.31 | 0.22 |
| Prevotellaceae bacterium DNF00733 | 0.42 | 0.10 |
| Endosymbiont of Onthophagus Taurus | 0.11 | 0.39 |
| Porphyromonas bennonis | 0.41 | 0.07 |
| Escherichia sp 3 26(2010) | 0.08 | 0.36 |
| Escherichia coli O127:H6 str E2348 69 | 0.35 | 0.05 |
| Porphyromonas uenonis | 0.35 | 0.01 |
| Streptococcus oralis | 0.02 | 0.33 |
| Mobiluncus curtisii subsp holmesii | 0.22 | 0.12 |
| Corynebacterium simulans | 0.12 | 0.22 |
| Prevotella sp S9 HS 17 | 0.10 | 0.24 |
| Moraxellaceae bacterium 4 m12 3 | 0.01 | 0.29 |
| Anaerococcus obesiensis ph10 | 0.24 | 0.02 |
| Arcanobacterium sp NML 06501 | 0.17 | 0.06 |
| Sphingomonas sp BAC260 | 0.00 | 0.22 |
| Escherichia coli CFT073 | 0.06 | 0.17 |
| Anaerococcus sp gpac028 | 0.20 | 0.02 |
| Peptoniphilus harei | 0.18 | 0.04 |
| Prevotella sp S8 F8 | 0.14 | 0.07 |
| Eubacterium sp oral clone BU061 | 0.06 | 0.15 |
| Facklamia hominis | 0.19 | 0.02 |
| Anaerococcus octavius | 0.12 | 0.08 |
| Clostridiales bacterium S5 A11 | 0.18 | 0.02 |
| Bacterium str Rauti | 0.12 | 0.06 |
| Peptoniphilus koenoeneniae | 0.15 | 0.02 |
| Porphyromonas sp S8 86 12 | 0.13 | 0.04 |
| Peptoniphilus sp PFC2 | 0.09 | 0.07 |
| Peptoniphilus sp oral taxon 836 | 0.12 | 0.04 |
| Anaerococcus tetradius | 0.11 | 0.04 |
| Propionibacterium sp V07 12348 | 0.07 | 0.07 |
| Actinomyces radingae | 0.11 | 0.02 |
| Mobiluncus curtisii subsp curtisii | 0.08 | 0.05 |
| Peptoniphilus sp 2002 38328 | 0.06 | 0.05 |
| Endosymbiont of Sphenophorus levis | 0.02 | 0.10 |
| Peptoniphilus sp 35 6 1 | 0.09 | 0.01 |
| Peptoniphilus sp CCUG 48151 | 0.04 | 0.06 |
| Peptoniphilus sp 1 1 | 0.05 | 0.02 |
| Peptoniphilus obesi ph1 | 0.03 | 0.01 |
| Unclassified species | 39.54 | 46.27 |

Abbreviations: **RA**=Relative Abundance.
